# BDNF/TrkB Signaling in Intracardiac Ganglia Modulates Cardiac Parasympathetic Tone

**DOI:** 10.3390/ijms27167403

**Published:** 2026-08-19

**Authors:** Jacopo Agrimi, Seungho Jun, Marie Anne Makoudjou, Roberto Luisetto, Lucia Bernardele, Giovanni Piccolo, Wenling Li, Elizabeth H. Smith, Megan D. Poston, Yoh-suke Mukouyama, Donald B. Hoover, Nazareno Paolocci

**Affiliations:** 1 Dipartimento di Scienze Biomediche, Università di Padova, 35131 Padova, Italy; marieanne.makoudjou@unipd.it (M.A.M.); lucia.bernardele@studenti.unipd.it (L.B.); giovanni.piccolo@unipd.it (G.P.); nazareno.paolocci@unipd.it (N.P.); 2 Department of Medicine, Johns Hopkins University School of Medicine, Baltimore, MD 21205, USA; seunghojun88@gmail.com; 3 DISCOG Department of Surgery, Oncology, and Gastroenterology, University of Padova, 35128 Padova, Italy; roberto.luisetto@unipd.it; 4 Laboratory of Stem Cell and Neuro-Vascular Biology, Cell and Developmental Biology Center, National Heart, Lung, and Blood Institute, Bethesda, MD 20892, USA; wenlingli@mail.nih.gov (W.L.); mukoyamay@nhlbi.nih.gov (Y.-s.M.); 5 Department of Biomedical Sciences, Quillen College of Medicine, East Tennessee State University, Johnson City, TN 37614, USA; lizsmith1489@gmail.com (E.H.S.); megan.poston@lmunet.edu (M.D.P.); hoover@mail.etsu.edu (D.B.H.); 6 Center of Excellence in Inflammation, Infectious Disease and Immunity, East Tennessee State University, Johnson City, TN 37614, USA

**Keywords:** BDNF, TrkB, intrinsic cardiac ganglia, intrinsic cardiac nervous system, autonomic nervous system, heart rate, satellite glial cells

## Abstract

Brain-derived neurotrophic factor (BDNF) impacts parasympathetic nervous system function by increasing the excitability of cardioinhibitory parasympathetic neurons in the brainstem, ultimately lowering heart rate (HR) and heightening resting parasympathetic tone. Yet, whether BDNF and its high-affinity receptor—tropomyosin receptor kinase B (TrkB)—also act more distally, i.e., at the level of cholinergic-sensitive intrinsic cardiac ganglia (ICGs), remains unclear. Hence, we conducted morphological and functional studies in neural crest-specific BDNF knockout mice (ncBDNF KO), a model that selectively ablates BDNF signaling in neural crest-derived autonomic structures, including the intrinsic cardiac nervous system. ncBDNF mice exhibited a significant rise in resting heart rate with unchanged baseline contractile performance, thus supporting the role of endogenous BDNF in maintaining physiological parasympathetic restraint. When examining the ICGs, immunofluorescence analysis revealed a highly compartmentalized organization, with BDNF being predominantly confined to cholinergic neuronal somata and TrkB mainly clustered instead in S100-positive satellite glial cells, thus unveiling a previously unrecognized neuron–glia BDNF/TrkB ICG pattern. Next, we directly infused BDNF in Langendorff-perfused isolated WT mouse hearts and observed a rapid and reproducible bradycardic response that was abrogated by atropine but potentiated by neostigmine, hence attesting to the cholinergic nature of such bradycardia. Of note, BDNF maintained its positive inotropic effects under muscarinic blockade, as witnessed by the enhanced left ventricular developed pressure, maximal dP/dt, and rate-pressure product, congruent with direct BDNF-evoked myocardial TrkB agonism. Thus, ICGs are additional relevant relay stations interposed between BDNF/TrkB signaling and parasympathetic modulation of heart function. Although through different molecular paths, BDNF-mediated modulation of ICG firing can coordinate with the previously reported BDNF positive inotropy/lusitropy to adapt cardiac performance to increased workload and/or stress conditions.

## 1. Introduction

The autonomic nervous system (ANS) plays a central role in cardiovascular homeostasis by continuously adjusting cardiac chronotropy, contractility, and conduction in response to increased workload or stress conditions [1]. The dynamic balance between sympathetic and parasympathetic activity contributes to cardiovascular homeostasis, whereas its disruption is a fomite of several cardiovascular disorders, including arrhythmias, heart failure, and sudden cardiac death [2,3,4,5].

Within the ANS, the intrinsic cardiac nervous system (ICNS) is a complex network of neurons and ganglionated plexi distributed throughout the heart to integrate sensory, sympathetic, and parasympathetic information, while generating local reflex responses that directly modulate sinoatrial node activity, atrioventricular conduction, and myocardial excitability [6,7,8,9]. Because of this integrative capacity, the ICNS has often been referred to as the heart’s “little brain” [8]. Importantly, structural and functional ICNS remodeling has been implicated in several pathological conditions, including myocardial infarction, heart failure, and cardiac arrhythmias [10,11,12,13].

The ICNS interconnected ganglia are anatomically designated as cardiac parasympathetic ganglia because they contain postganglionic parasympathetic neurons that provide cholinergic innervation of the heart [14,15]. However, most intrinsic cardiac neurons exhibit a complex neurochemical phenotype, including markers of peptidergic, noradrenergic, and nitrergic neurons, as well as neurotrophin (NT)-associated signaling via tropomyosin receptor kinase A (TrkA) and p75 NT receptors [16], in addition to the expected cholinergic markers [17,18,19,20,21,22,23]. Moreover, in vivo studies have documented that the intrinsic cardiac ganglia contain noradrenergic efferent neurons, local circuit neurons, and sensory neurons [24,25].

Although models are used frequently in cardiovascular research, very little is known about mouse intrinsic cardiac neurons. Accordingly, our group and others have pioneered work showing that neurons from the adult mouse heart could be maintained in culture, while defining their basic phenotypic properties. Neurons in culture are primarily unipolar, and 89% had prominent neurite outgrowth after 3 days, with many neurites forming close appositions with other neurons and non-neuronal cells [26]. Neurite outgrowth is drastically reduced when neurons are kept in culture with a majority of nonneural cells eliminated, suggesting that non-neuronal cells release molecules that support neurite outgrowth. All neurons in coculture showed immunoreactivity for a full complement of cholinergic markers, but about 21% also stained for tyrosine hydroxylase, as observed previously in sections of intrinsic cardiac ganglia from mice and humans [26]. Thus, these cultures provide a viable model for evaluating the physiology, pharmacology, and sensitivity to trophic factors of adult mouse cardiac parasympathetic neurons.

Brain-derived neurotrophic factor (BDNF) is a major regulator of neuronal survival, synaptic plasticity, and neurotransmission through activation of its high-affinity receptor TrkB [27,28,29]. BDNF has emerged as an important modulator of autonomic circuits. Notably, Wan and colleagues demonstrated that reduced BDNF signaling increases resting heart rate by impairing parasympathetic activity, while central BDNF administration restores vagal chronotropic control by enhancing the excitability of cardioinhibitory brainstem neurons [30]. These findings established BDNF as a key regulator of cardiac parasympathetic tone. In parallel, myocardial BDNF–TrkB signaling has also been implicated in modulating myocardial contractility/relaxation [31], as well as in the myocyte response to physiological [32] and pathological (ischemic) stress [33]. Recent evidence further suggests that NT signaling may contribute to neuro-glial communication within peripheral autonomic circuits. Glial cells actively produce neurotrophic factors and express NT receptors, supporting neuronal maintenance and synaptic plasticity [34,35,36]. In the ICNS, NGF-expressing glial populations have been identified in nodal regions, raising the possibility that local neurotrophic signal and satellite glial cells are the primary TrkB-responsive compartment, thereby contributing to intracardiac circuit organization and function [37]. In stark contrast, whether BDNF/TrkB signaling is present at the ICG level and what role it plays remains unknown. In more detail, it remains to be determined whether local BDNF–TrkB signaling contributes to neuron–glia communication to directly modulate cardiac chronotropy independently of central autonomic pathways.

Against this background, we combined genetic, anatomical, and ex vivo functional approaches to test the hypothesis that intracardiac ganglia contain an intrinsic BDNF-responsive neuro-glial circuit. Our findings identify a previously unrecognized compartmentalized BDNF–TrkB signaling architecture in which cholinergic neurons act as a local source of BDNF, and satellite glial cells are the primary TrkB-responsive compartment, thereby establishing the ICNS as a peripheral effector site for BDNF-dependent parasympathetic control of heart rate.

## 2. Results

### 2.1. Neural Crest-Specific BDNF Deletion Preserves Baseline Cardiac Function but Increases Resting Heart Rate

To investigate the physiological relevance of endogenous autonomic BDNF in cardiac regulation, we studied neural crest-specific BDNF knockout mice (ncBDNF KO), generated by crossing Bdnf^2lox^ mice with Wnt1-Cre2 transgenic mice (Tables S1–S3), thereby selectively deleting BDNF in neural crest-derived autonomic lineages. This model targets both peripheral autonomic structures, including intracardiac ganglia, and central neural crest-derived components, thus extending previous observations obtained in global BDNF-deficient models [30].

Comprehensive echocardiographic assessment revealed no significant differences in baseline cardiac function between ncBDNF KO mice and littermate controls. Parameters reflecting global myocardial performance, including heart rate-independent functional indices and overall contractile performance (Table 1), were preserved, indicating that autonomic BDNF is not required for basal myocardial function under resting conditions.

**Figure 1:**
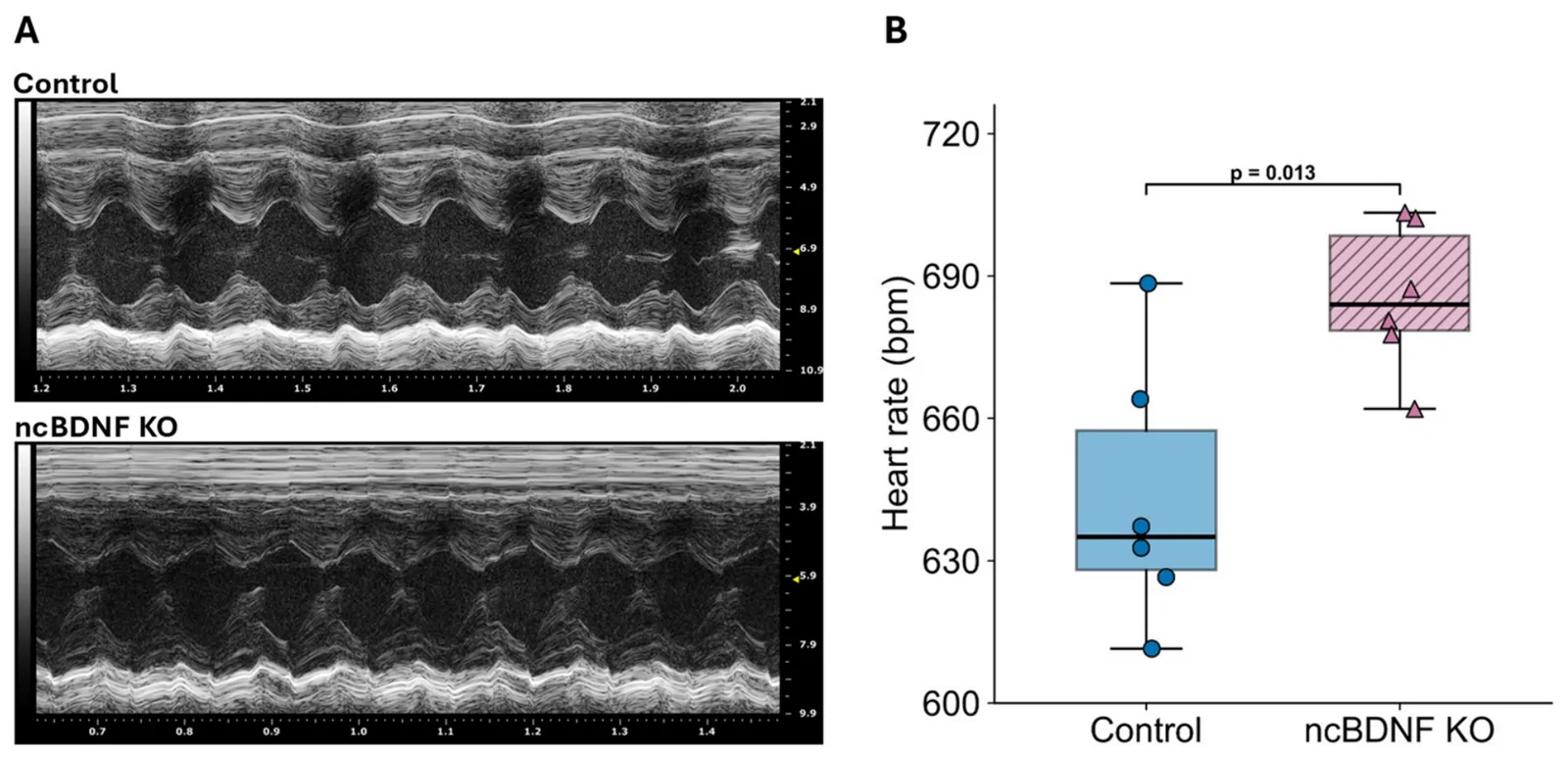
Neural crest-specific BDNF deletion increases resting heart rate without affecting baseline cardiac function. (**A**) Representative M-mode echocardiographic recordings from control and neural crest-specific BDNF knockout (ncBDNF KO) mice. (**B**) Quantification of the resting heart rate. Data are shown as box-and-whisker plots with individual values, n = 6. Statistical comparisons were performed using unpaired two-tailed Student’s *t*-test (Welch’s correction where appropriate). *p* < 0.05 was considered statistically significant.

In contrast, rhythm analysis revealed a significant increase in resting heart rate in ncBDNF KO mice compared to controls (Figure 1B). This chronotropic alteration occurred in the absence of detectable systolic dysfunction, suggesting a selective disruption of autonomic regulation rather than a primary myocardial defect.

These findings are consistent with previous evidence showing that BDNF enhances parasympathetic tone through central vagal circuits [30] and further supports a physiological role for endogenous BDNF in maintaining resting vagal restraint over cardiac chronotropy.

### 2.2. BDNF–TrkB Signaling Is Organized Within a Neuro-Glial Synaptic Architecture in Intracardiac Ganglia

**Figure 2:**
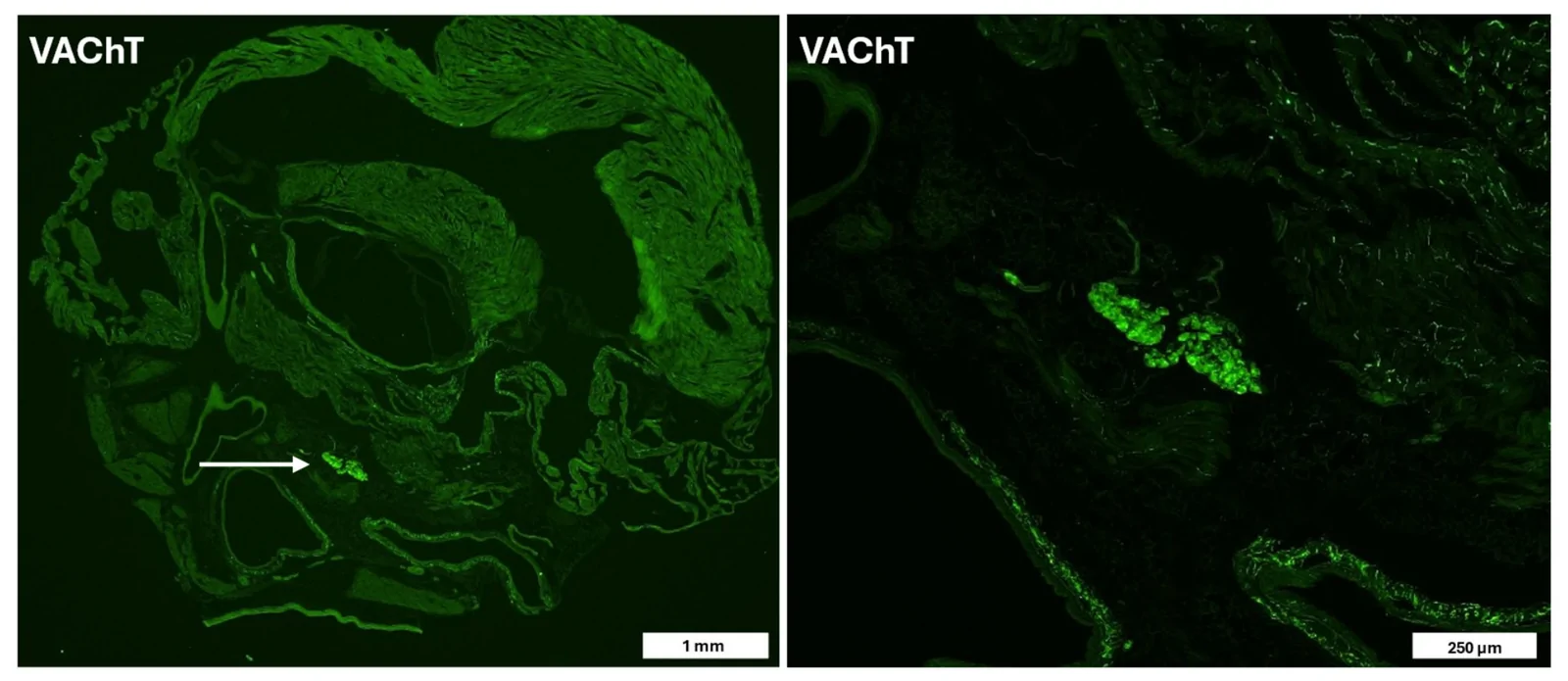
VAChT immunostaining identifies cholinergic intracardiac ganglia in adult mouse heart. Representative low- and high-magnification images of cardiac sections immunolabeled for vesicular acetylcholine transporter (VAChT), a marker of cholinergic neurons. Low-magnification imaging (**left**) shows the anatomical localization of an intracardiac ganglion (arrow) within the epicardial fat at the atrial base. Higher magnification (**right**) reveals clustered VAChT-positive neuronal somata within the ganglion, confirming the cholinergic identity of intrinsic cardiac neurons. Scale bars: 1 mm (**left**), 250 μm (**right**).

To investigate whether the BDNF–TrkB signaling axis is anatomically represented within the intrinsic cardiac nervous system (ICNS), we first identified intracardiac ganglia in adult mouse hearts using the vesicular acetylcholine transporter (VAChT), a marker of cholinergic neurons. Low-magnification imaging localized discrete VAChT-positive ganglionic structures within cardiac tissue, whereas higher magnification revealed clusters of VAChT-immunoreactive neuronal somata (Figure 2). These observations confirmed the cholinergic identity of the intracardiac neuronal structures subsequently examined for neurotrophin signaling.

**Figure 3:**
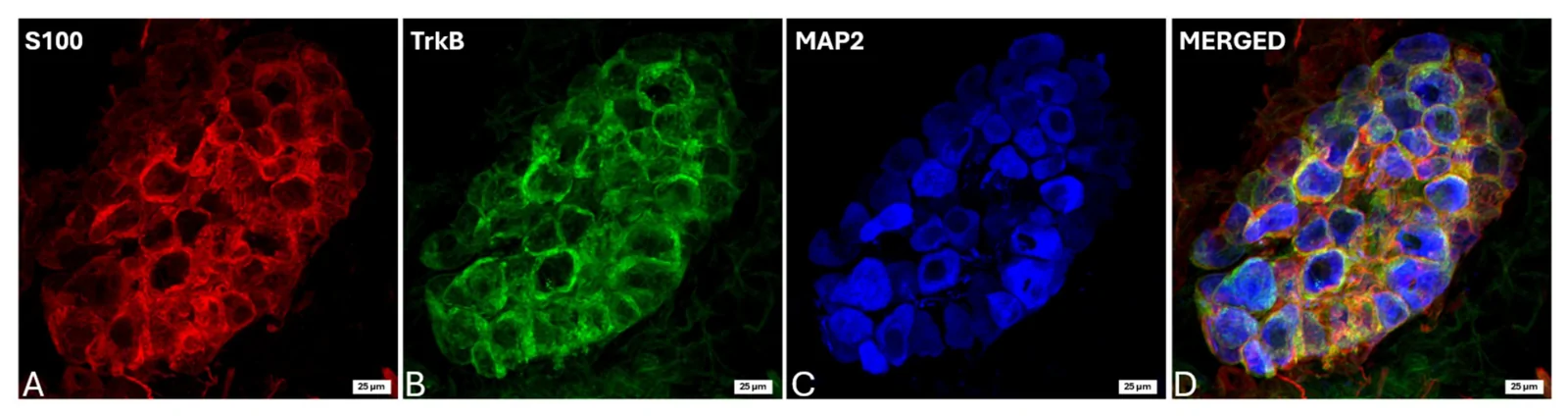
TrkB is predominantly associated with the satellite glial compartment of murine intracardiac ganglia. Representative immunofluorescence images of an intracardiac ganglion labeled for the satellite glial marker S100 (**A**), TrkB (**B**), and the neuronal marker MAP2 (**C**), with merged channels shown in (**D**). TrkB immunoreactivity displays a prominent perineuronal distribution and is predominantly associated with the S100-positive glial network surrounding MAP2-positive neuronal somata.

We next investigated the cellular distribution of TrkB within intracardiac ganglia. Immunofluorescence analysis revealed robust TrkB immunoreactivity throughout the ganglionic compartment, displaying a prominent perineuronal pattern closely resembling the organization of S100-positive satellite glial cells (Figure 3). Multiplex labeling for S100, TrkB, and the neuronal marker MAP2 further demonstrated that TrkB immunoreactivity was predominantly associated with the S100-positive glial network surrounding MAP2-positive neuronal somata. This spatial organization identifies satellite glial cells as a major TrkB-expressing cellular compartment within murine intracardiac ganglia.

**Figure 4:**
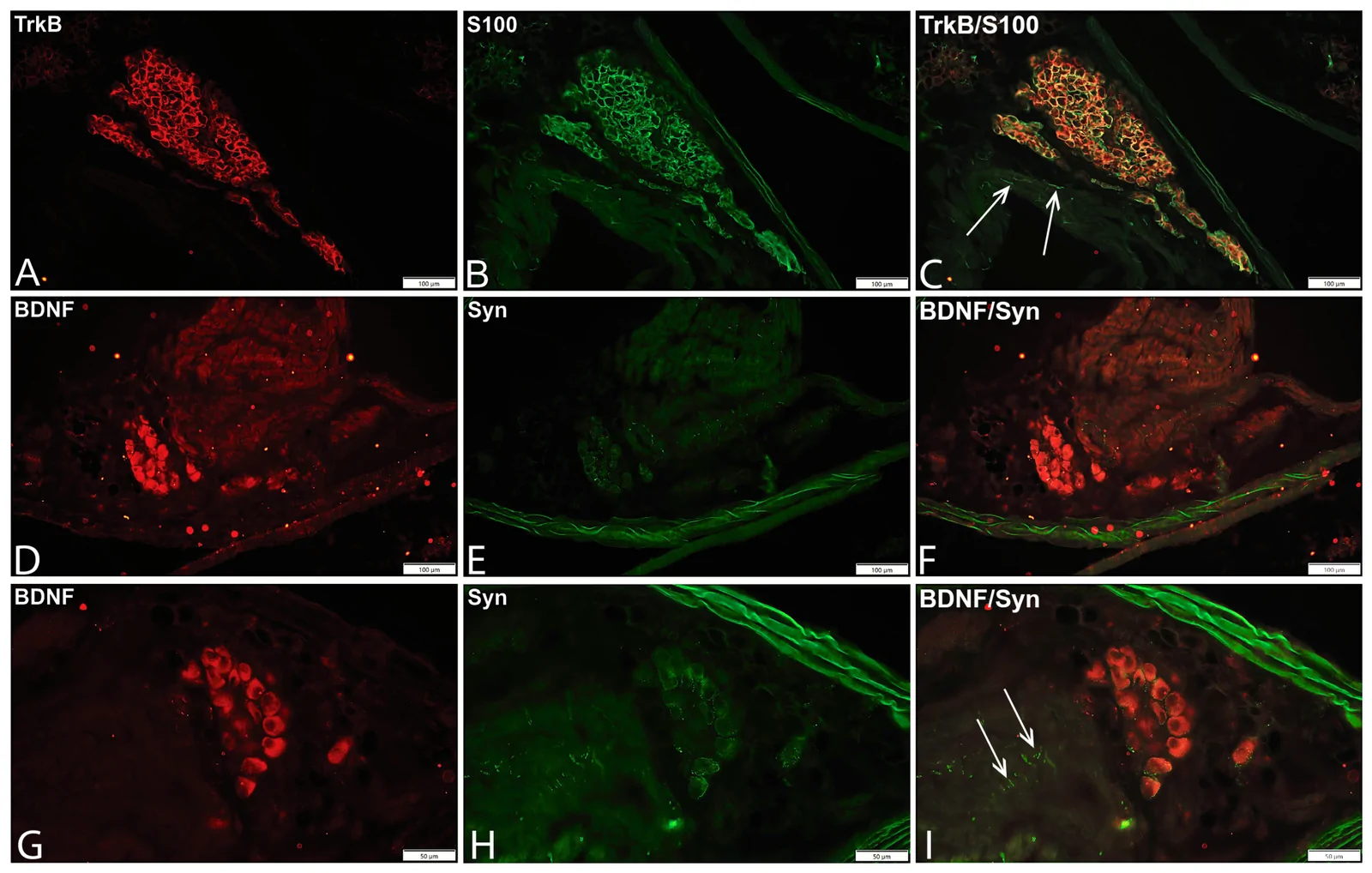
BDNF-positive neurons reside within a synaptically organized intracardiac ganglionic microenvironment. Representative immunofluorescence images of murine intracardiac ganglia labeled for TrkB (**A**), S100 (**B**), BDNF (**D**,**G**), and the presynaptic vesicle-associated protein synaptophysin (Syn; (**E**,**H**)). TrkB and S100 display similar perineuronal distribution patterns within ganglionic structures (**C**), whereas BDNF immunoreactivity is predominantly localized within neuronal somata (**D**,**G**). Double immunolabeling for BDNF and the synaptic vesicle-associated protein synaptophysin revealed distinct localization patterns within intracardiac ganglia (**F**,**I**). Synaptophysin immunoreactivity was observed in presumed presynaptic processes surrounding BDNF-positive neuronal cell bodies (**F**,**I**). Synaptophysin-positive nerve fibers were also detected in the adjacent atrial tissue (panel (**I**), white arrows), where BDNF immunoreactivity was absent. Similarly, TrkB immunoreactivity was not detected in atrial Schwann cells (panel (**C**), white arrows) or nerve fibers.

In contrast, BDNF immunoreactivity exhibited a distinct cellular distribution and was predominantly detected within ganglionic neuronal somata (Figure 4). This neuronal pattern of BDNF expression was consistently observed across intracardiac ganglionic structures and was spatially complementary to the perineuronal TrkB/S100-positive compartment. Thus, BDNF-positive neuronal cell bodies appear embedded within a TrkB-positive glial network, revealing a highly compartmentalized organization of local neurotrophin signaling. Double labeling for BDNF and synaptophysin, a synaptic vesicle-associated protein, showed separate localization within the ganglia. Synaptopohysin was localized to presumed presynaptic processes surrounding BDNF+ neurons. Synaptophysin was also detected in nerve fibers in nearby atrium (Figure 4I, white arrows), which also lacked BDNF. Staining for TrkB was likewise absent in atrial nerve fibers and associated S100-positive Schwann cells (Figure 4C, white arrows).

Collectively, these findings reveal a spatially organized neuro–glial architecture of BDNF–TrkB signaling within intracardiac ganglia, in which neuronal somata represent a prominent local source of BDNF and surrounding S100-positive satellite glial cells constitute a major TrkB-expressing compartment. The close anatomical relationship between these cellular domains and the local synaptic environment supports the existence of a compartmentalized BDNF-responsive neuro–glial signaling unit within the ICNS.

### 2.3. Exogenous BDNF Induces Bradycardia in Isolated Perfused Hearts Independently of Central Autonomic Inputs

Having established that endogenous autonomic BDNF contributes to the regulation of resting heart rate in vivo and identified a local BDNF–TrkB signaling architecture within intracardiac ganglia, we next asked whether BDNF is sufficient to directly modulate cardiac chronotropy at the level of the heart itself.

**Figure 5:**
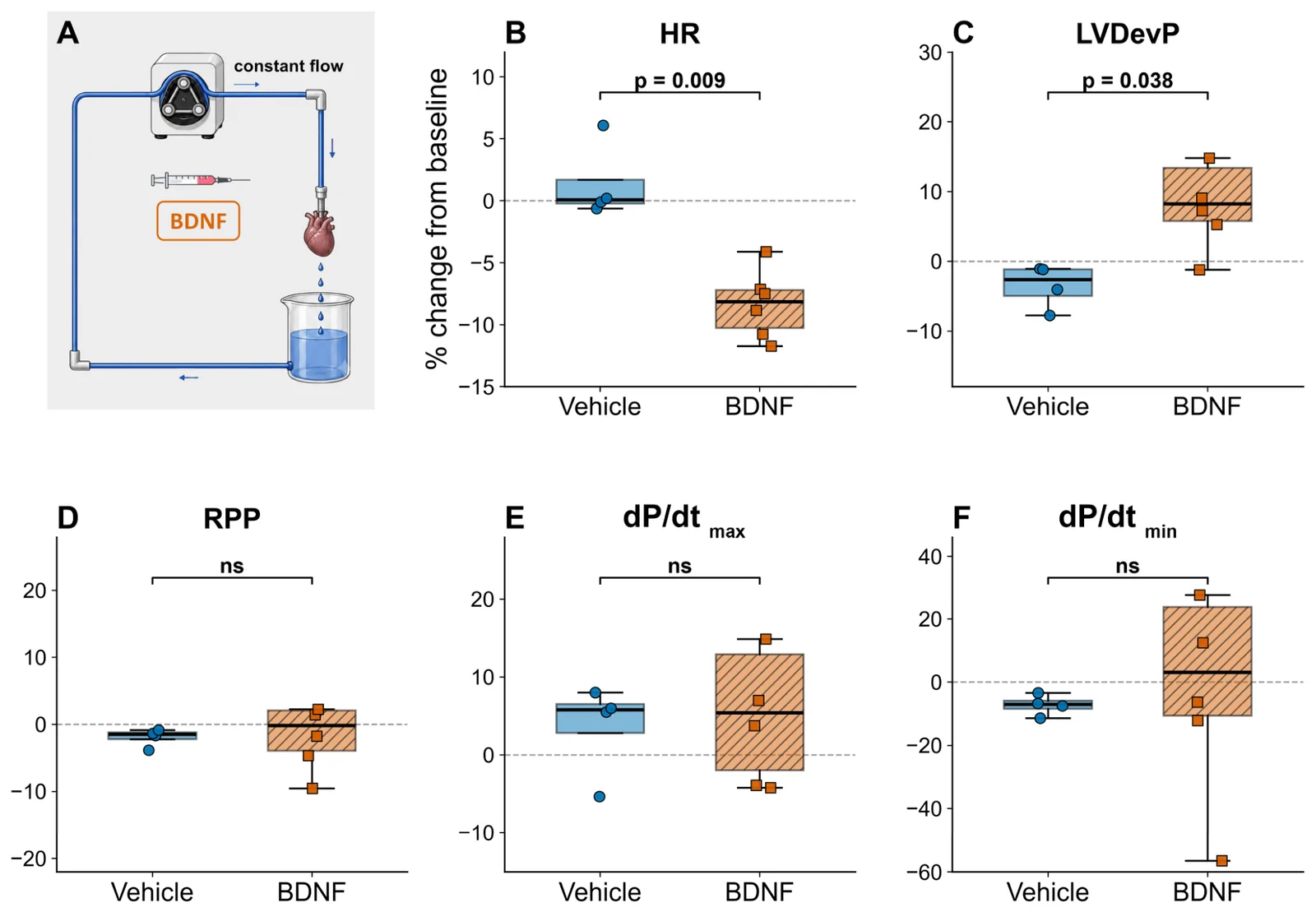
Acute BDNF infusion modulates cardiac function in isolated perfused hearts. (**A**) Isolated-perfused heart scheme, BDNF or vehicle administration. (**B**) Heart rate (HR); (**C**) left ventricular developed pressure (LVDevP); (**D**) rate-pressure product (RPP), expressed as percentage change from baseline, was not significantly altered by BDNF; (**E**) maximal dP/dt, expressed; and (**F**) minimal dP/dt. All parameters expressed as percentage change from baseline after 10 min from infusion of BDNF or Vehicle. Data are shown as box-and-whisker plots with individual values, n = 4–6. Comparisons between groups were performed using the non-parametric Mann–Whitney *U* test. Statistical significance is indicated in the individual panels.

To address this question, we performed Langendorff perfusion experiments in isolated mouse hearts, a preparation that preserves the intrinsic cardiac nervous system while excluding all central and systemic autonomic influences (Figure 5). This approach allowed us to specifically interrogate whether BDNF can act locally within the neurocardiac compartment.

Acute infusion of recombinant BDNF (10 nM, 10 min) induced a rapid and significant reduction in heart rate compared to baseline and vehicle-treated controls (Figure 5A). The chronotropic response was reproducible across preparations and occurred within minutes of BDNF administration, demonstrating that BDNF is sufficient to acutely modulate cardiac rhythm in the intact ex vivo heart.

Importantly, because these experiments were performed in the complete absence of brainstem vagal input and circulating neurohumoral factors, the observed bradycardic response cannot be attributed to central parasympathetic mechanisms previously described [30]. Instead, these findings provide direct evidence that the heart contains an intrinsic BDNF-responsive autonomic circuit capable of locally regulating chronotropy.

Together, these results identify the intrinsic cardiac nervous system as a previously unrecognized effector site for BDNF-dependent parasympathetic modulation and suggest that local BDNF signaling may complement central autonomic pathways in the physiological control of heart rate.

### 2.4. BDNF-Induced Bradycardia Depends on Local Cholinergic Transmission While Preserving Direct Myocardial Inotropic Effects

To define the mechanism underlying the negative chronotropic effect of BDNF, we investigated whether local cholinergic signaling within intracardiac ganglia mediates this response. Given that intracardiac ganglia contain cholinergic postganglionic neurons projecting to the sinoatrial node, we hypothesized that BDNF-induced bradycardia might result from enhanced parasympathetic neurotransmission.

**Figure 6:**
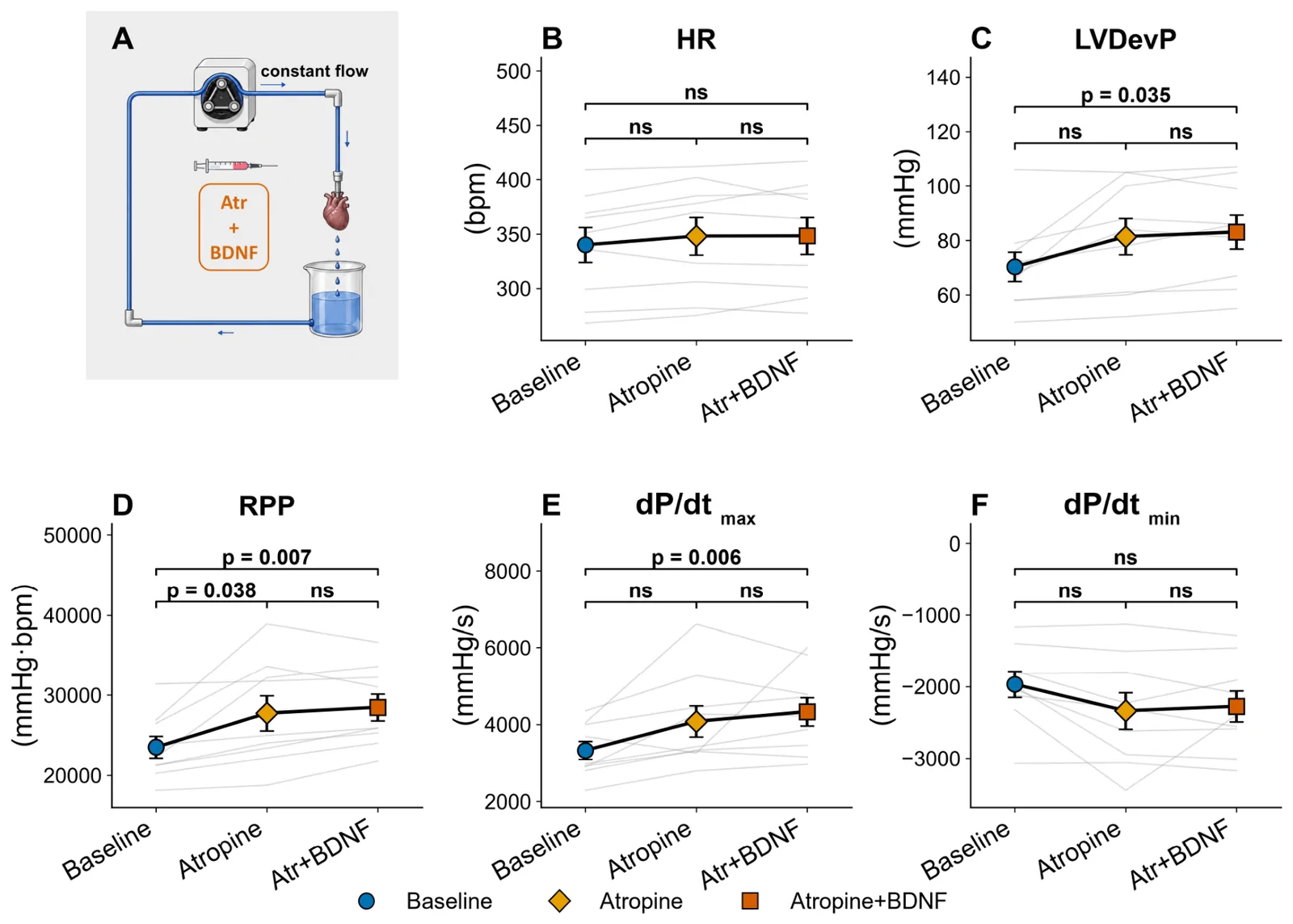
Muscarinic blockade abolishes the chronotropic effect of BDNF while not affecting its positive inotropic actions in isolated perfused hearts. (**A**) Experimental setting: Langendorff-perfused mouse hearts (constant flow) and protocols (baseline, atropine, and BDNF administration). (**B**) Heart rate (HR); (**C**) left ventricular developed pressure (LVDevP); (**D**) rate-pressure product (RPP); (**E**) maximal dP/dt and (**F**) minimal dP/dt, at baseline, after atropine, and after atropine + BDNF. Data are shown as paired individual values (gray lines) with mean ± SEM, n = 8. Comparisons across experimental conditions were performed using a non-parametric repeated-measures ANOVA (Friedman test). Statistical significance is indicated in the individual panels.

To test this hypothesis, isolated hearts were co-perfused with BDNF and atropine, a competitive muscarinic receptor antagonist. Pharmacological blockade of muscarinic signaling completely abolished the reduction in heart rate induced by BDNF alone (Figure 6B), with heart rate remaining comparable to baseline values throughout the recording period. These findings demonstrate that muscarinic receptor activation is necessary for the negative chronotropic action of BDNF and strongly indicate that this effect depends on intact cholinergic neurotransmission downstream of intracardiac ganglionic activation.

**Figure 7:**
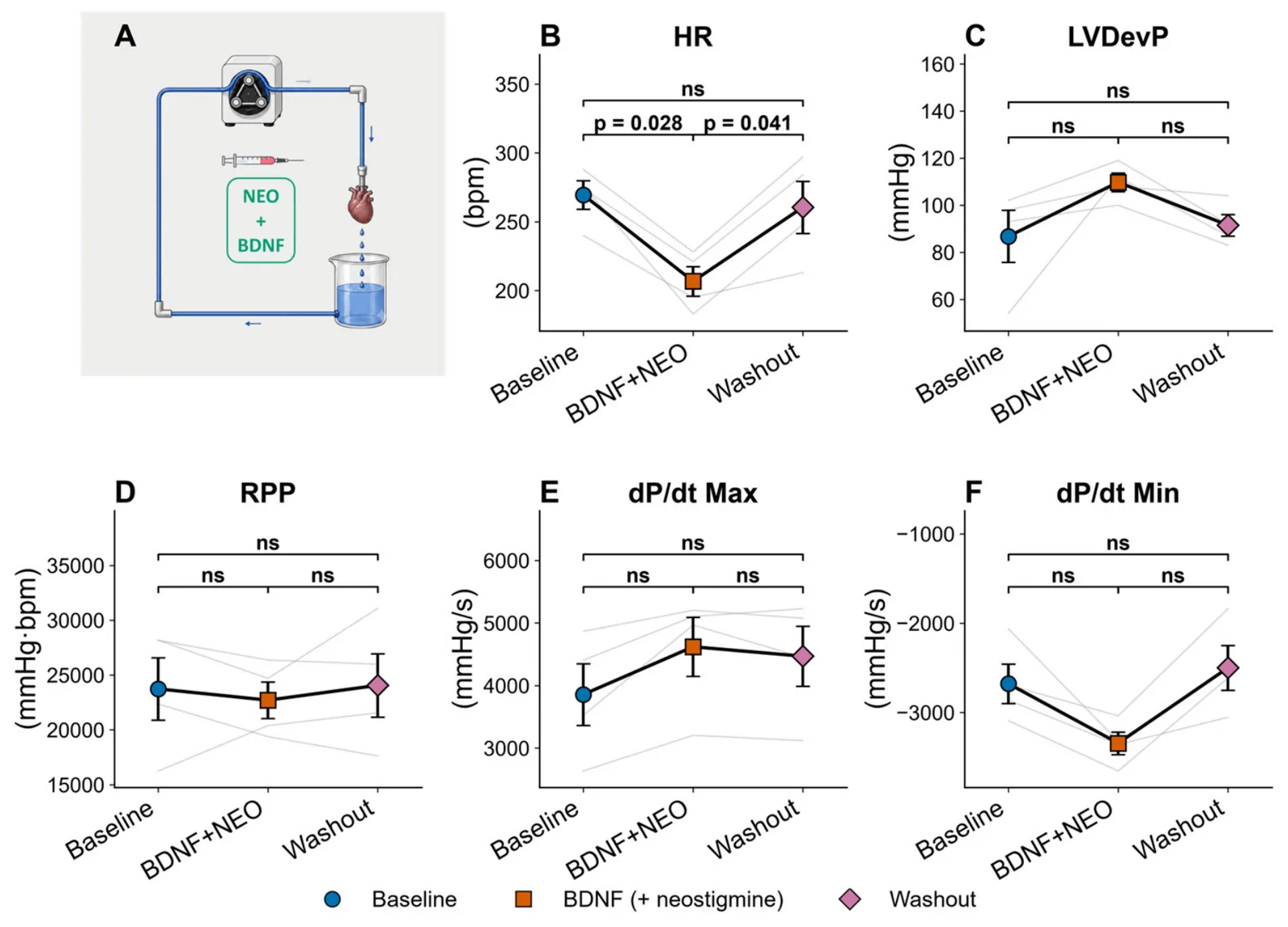
Cholinesterase inhibition potentiates the bradycardic effect of BDNF in isolated perfused hearts. (**A**) Isolated-perfused heart scheme, neostigmine and BDNF administration. (**B**) Heart rate (HR); (**C**) left ventricular developed pressure (LVDevP); (**D**) rate-pressure product (RPP); (**E**) maximal dP/dt; (**F**) minimal dP/dt. Data are shown as paired individual values (gray lines) with mean ± SEM, n = 5. Comparisons across experimental conditions were performed using a non-parametric repeated-measures ANOVA (Friedman test). Statistical significance is indicated in the individual panels.

To further evaluate this interpretation, we next enhanced cholinergic tone by co-infusing BDNF with neostigmine, an acetylcholinesterase inhibitor that prolongs acetylcholine availability at synaptic clefts. Under these conditions, BDNF-induced bradycardia was significantly potentiated and prolonged compared to BDNF alone (Figure 7). Both the magnitude and duration of the chronotropic response were enhanced, consistent with amplification of parasympathetic neurotransmission. Because these experiments were performed in isolated perfused hearts, these findings further support a mechanism in which BDNF acts upstream of muscarinic receptor activation, likely at the level of intracardiac ganglia, to facilitate local cholinergic output.

In contrast to its chronotropic effects, indices of myocardial contractility remained responsive to BDNF even in the presence of atropine. Left ventricular developed pressure (LVDevP) increased significantly following BDNF infusion, while both maximal dP/dt and rate-pressure product (RPP) were robustly enhanced despite muscarinic blockade (Figure 6D,E). These observations indicate that the positive inotropic actions of BDNF are mechanistically independent of cholinergic signaling. This functional dissociation is consistent with our previous evidence showing direct TrkB-dependent signaling in cardiomyocytes and supports a dual compartmental model of BDNF action in the heart. In this model, BDNF acts locally within intracardiac ganglia to enhance parasympathetic cholinergic signaling and reduce heart rate, while simultaneously acting directly on myocardial cells to increase contractile performance.

Collectively, these findings support the concept that BDNF exerts distinct neurocardiac actions through anatomically and functionally segregated pathways. However, the cellular mechanisms linking neuronal BDNF to enhanced cholinergic output remain to be directly established.

## 3. Discussion

The present study identifies a previously unrecognized local BDNF–TrkB signaling circuit within intracardiac ganglia and provides evidence that this pathway contributes to cardiac parasympathetic regulation. Although BDNF has previously been implicated in central autonomic control by enhancing the excitability of brainstem cardioinhibitory neurons [19], whether BDNF also acts at the level of the intrinsic cardiac nervous system (ICNS) remained unknown. Our findings extend this concept by demonstrating that intracardiac ganglia constitute a peripheral neurotrophic signaling hub in which BDNF locally modulates cardiac chronotropy. Specifically, we show that neural crest-derived BDNF deficiency is associated with increased resting heart rate, that BDNF and TrkB are compartmentalized within a neuron–glia signaling architecture in intracardiac ganglia, and that acute exogenous BDNF is sufficient to induce a cholinergic-dependent bradycardic response in isolated perfused hearts. Together, these findings position the ICNS as a novel effector site for BDNF-dependent autonomic regulation.

The in vivo phenotype observed in neural crest-specific BDNF knockout mice supports the physiological relevance of endogenous BDNF in maintaining basal cardiac vagal restraint. Mutant animals exhibited elevated resting heart rate in the absence of detectable baseline systolic dysfunction, consistent with previous reports in global BDNF-deficient mice [30]. An important aspect of the in vivo phenotype is the clear dissociation between chronotropic regulation and basal myocardial performance. Despite the significant increase in resting heart rate, ncBDNF KO mice displayed preserved systolic function and normal cardiac morphology. This observation suggests that endogenous neural crest-derived BDNF primarily contributes to autonomic regulation of cardiac rhythm rather than to the maintenance of basal myocardial contractility. These findings are consistent with the concept that parasympathetic modulation predominantly affects chronotropy under physiological conditions, whereas intrinsic ventricular contractile performance remains largely preserved in the absence of overt cardiac stress.

An important limitation of this model must be acknowledged. Wnt1-Cre drives recombination not only in pre-migratory neural crest derivatives but also in embryonic midbrain and hindbrain regions, including structures potentially involved in central autonomic regulation. Therefore, the tachycardic phenotype observed in vivo cannot be exclusively attributed to loss of BDNF within peripheral autonomic structures or the ICNS itself. This caveat is particularly relevant given the established role of BDNF in vagal brainstem circuits. In this context, our ex vivo experiments are critical, as they directly isolate the heart from central and systemic autonomic influences and provide causal evidence of a local cardiac mechanism. Accordingly, the in vivo and ex vivo findings should be viewed as complementary rather than redundant. While the in vivo model demonstrates the physiological relevance of neural crest-derived BDNF for cardiac autonomic regulation, the isolated heart preparation specifically establishes that BDNF is also capable of modulating cardiac chronotropy through a local intracardiac mechanism, independently of central autonomic input.

One of the most striking findings of this study is the highly compartmentalized cellular organization of BDNF and TrkB within intracardiac ganglia. While BDNF was predominantly localized in cholinergic neuronal somata, TrkB expression was largely confined to S100-positive satellite glial cells. This anatomical arrangement suggests the existence of a local paracrine neuron–glia signaling unit, rather than a classical neuron-autonomous neurotrophic pathway. Satellite glial cells are increasingly recognized as active regulators of autonomic ganglionic function, sharing functional similarities with astrocytes in the central nervous system [38,39]. They regulate extracellular potassium buffering, neurotransmitter turnover, calcium signaling, and neuroinflammatory responses, thereby directly influencing neuronal excitability and synaptic efficacy. Our findings now place BDNF–TrkB signaling within this emerging neuro-glial framework of cardiac autonomic regulation.

Functionally, the Langendorff experiments provide direct evidence that BDNF can acutely modulate cardiac chronotropy independently of the central nervous system. Acute BDNF infusion induced a rapid and reproducible bradycardic response, demonstrating that the intact heart contains a local BDNF-responsive circuit capable of influencing rhythm generation. Importantly, this effect was completely abolished by atropine, establishing that muscarinic receptor activation is required for BDNF-mediated chronotropic control. Since atropine acts downstream of acetylcholine release at the effector level, these data strongly indicate that BDNF acts upstream of muscarinic signaling by facilitating local parasympathetic cholinergic output.

The potentiation of BDNF-induced bradycardia by neostigmine further strengthens this interpretation. Neostigmine prolongs acetylcholine availability by inhibiting acetylcholinesterase, thereby amplifying cholinergic signaling at neuroeffector junctions. The enhanced magnitude and duration of the BDNF response during cholinesterase inhibition strongly suggest that BDNF increases the efficacy of acetylcholine-dependent transmission rather than acting directly on the sinoatrial node. Within intracardiac ganglia, postganglionic parasympathetic neurons release acetylcholine through a classical vesicular calcium-dependent mechanism involving choline acetyltransferase (ChAT) and the vesicular acetylcholine transporter (VAChT), ultimately acting on M2 muscarinic receptors in the sinoatrial node. Our pharmacological and immunohistochemical findings place BDNF functionally upstream of this machinery, suggesting a modulatory role at the ganglionic level.

**Figure 8:**
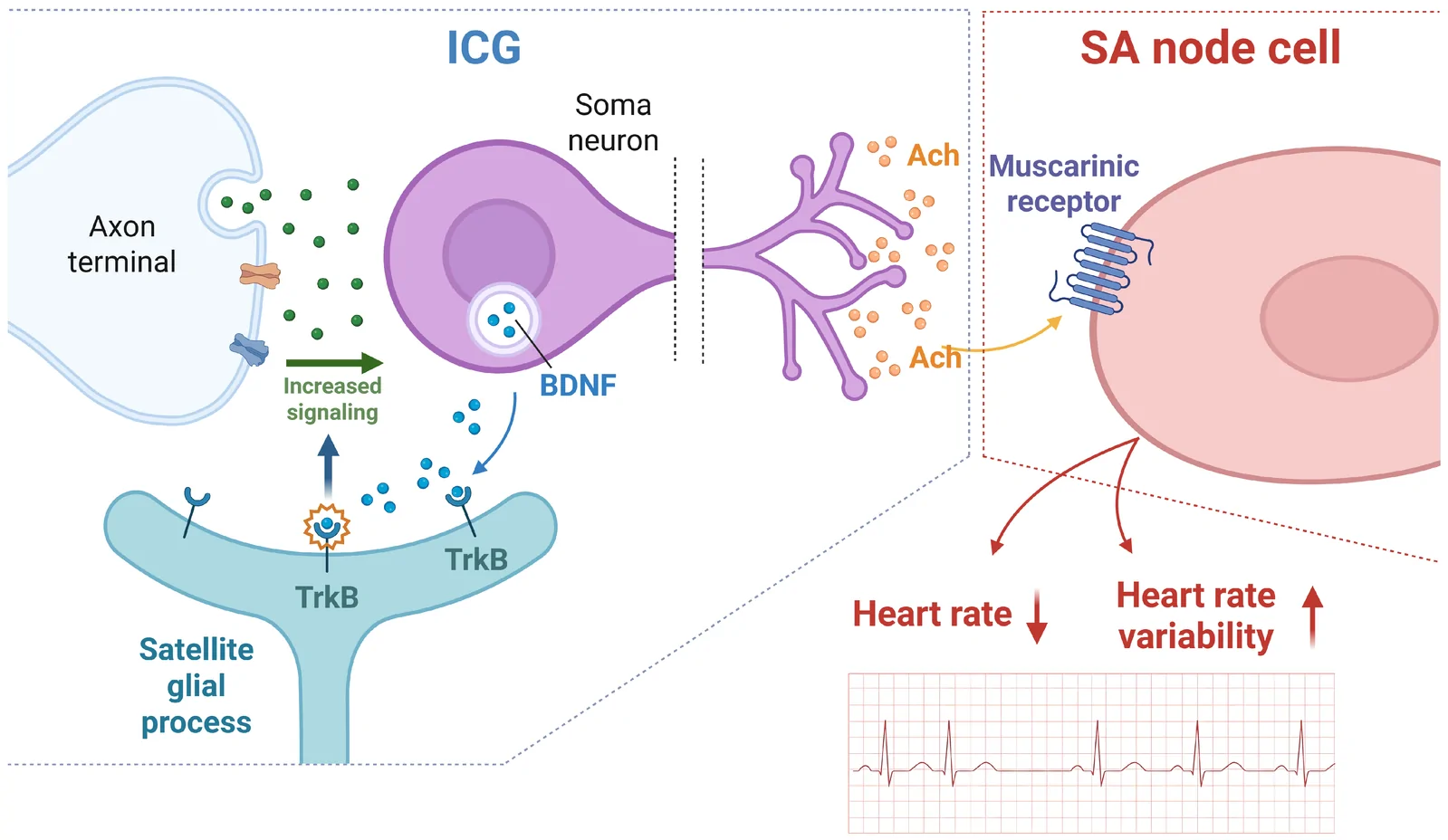
Conceptual model for BDNF-dependent neuro-glial modulation of parasympathetic signaling within intracardiac ganglia. Within intracardiac ganglia (ICG), BDNF released by cholinergic neurons is proposed to act locally on TrkB-expressing satellite glial cells, establishing a tripartite neuro-glial signaling unit. Through TrkB-dependent mechanisms, glial cells may modulate the ganglionic microenvironment by regulating ionic homeostasis, gliotransmitter release, metabolic support, or secondary trophic signaling, ultimately facilitating acetylcholine (ACh) release from postganglionic parasympathetic neurons. Increased cholinergic transmission at the neuroeffector junction enhances activation of muscarinic receptors in sinoatrial node pacemaker cells, resulting in reduced heart rate and increased heart rate variability.

A mechanistic question arising from our anatomical and pharmacological observations is how neuronal BDNF and glial TrkB signaling could facilitate cholinergic output. Previous studies in other cholinergic systems, including the neuromuscular junction, have shown that BDNF enhances acetylcholine release through TrkB-dependent activation of presynaptic signaling cascades involving PLCγ and PKC, increasing transmitter release probability and synaptic efficacy [40]. However, the compartmentalized architecture observed here suggests a distinct mechanism in the ICNS. We propose that BDNF released by intracardiac cholinergic neurons acts on neighboring TrkB-positive satellite glial cells, which in turn modulate the ganglionic microenvironment to enhance neuronal excitability or transmitter release. This could involve regulation of extracellular potassium, calcium-dependent gliotransmitter release, ATP/adenosine signaling, metabolic support, or secondary trophic signaling. Such a model is consistent with the emerging concept of tripartite neuro-glial synapses within cardiac ganglia [39,41] and provides a plausible mechanism linking local neurotrophic signaling to parasympathetic output (Figure 8). However, the present study does not directly demonstrate BDNF release, TrkB activation in satellite glial cells, or glia-to-neuron signaling. Therefore, the mechanism proposed below should be regarded as a biologically plausible working hypothesis that integrates the current observations and provides a framework for future mechanistic investigations.

In parallel to its autonomic effects, our data also reinforces the concept of direct myocardial BDNF signaling. The positive inotropic effects of BDNF persisted despite muscarinic blockade, indicating that BDNF enhances myocardial contractility through a mechanism independent of cholinergic transmission. This observation is fully consistent with previous evidence demonstrating direct TrkB signaling in ventricular cardiomyocytes and supports a dual-compartment model of BDNF action in the heart [31,33]. In this model, BDNF simultaneously modulates cardiac function through two anatomically and mechanistically distinct pathways: a local neuro-glial parasympathetic circuit controlling chronotropy and a direct myocardial signaling axis enhancing contractility.

These findings may have broader implications for cardiac disease. Intracardiac ganglionic remodeling is increasingly recognized as an important contributor to arrhythmogenesis, heart failure progression, and autonomic imbalance [10,12,13]. A neurotrophic signaling mechanism that modulates local parasympathetic tone introduces a new layer of plasticity within the ICNS and may represent an adaptive or maladaptive response under pathological conditions. Altered BDNF signaling has been implicated in heart failure, ischemia, and autonomic dysfunction, raising the possibility that disruption of this local neuron–glia axis could contribute to disease-associated autonomic remodeling.

Several limitations should be considered. First, the developmental and central recombination pattern of Wnt1-Cre limits the anatomical specificity of the in vivo knockout model. Second, while our pharmacological experiments strongly support enhanced cholinergic output, we did not directly measure acetylcholine release or ganglionic neuronal firing. Third, the functional contribution of satellite glial TrkB signaling remains inferential and will require direct validation. Future studies combining ganglionic electrophysiology, calcium imaging, local TrkB antagonism, and conditional glial-specific manipulations will be necessary to dissect the precise mechanisms involved.

In conclusion, this study identifies intracardiac ganglia as a previously unrecognized site of BDNF-dependent parasympathetic regulation and reveals a novel neuron–glia trophic signaling architecture within the ICNS. These findings expand the current understanding of cardiac autonomic regulation and establish BDNF as a dual neurocardiac modulator acting both at the level of local autonomic circuits and directly within the myocardium.

## 4. Materials and Methods

### 4.1. Animals and Housing

Mice were housed in a specific pathogen-free animal facility under controlled environmental conditions (22 ± 2 °C; 50–60% humidity) on a 12 h light/12 h dark cycle, with free access to standard laboratory chow and water. Animals were group-housed whenever possible and monitored daily for health status and welfare. All experiments were performed in adult mice aged 12–16 weeks. Experimental procedures were conducted in accordance with institutional guidelines and national regulations governing the care and use of laboratory animals and were approved by Johns Hopkins University Animal Care and Use Committee (protocol number MO23M237, approval date 18 November 2024).

### 4.2. Generation of Neural Crest-Specific BDNF Knockout Mice

Neural crest-specific BDNF knockout (ncBDNF KO) mice were generated by crossing *Bdnf*^flox/flox^ mice with *Wnt1*-Cre2 transgenic mice. The *Bdnf*^flox/flox^ mouse line carries loxP sites flanking the coding region of the *Bdnf* gene, allowing Cre recombinase-mediated excision of the gene in a tissue-specific manner while preserving normal BDNF expression in the absence of Cre. Homozygous *Bdnf*^flox/flox^ mice are viable and fertile. The *Wnt1*-Cre2 transgenic line expresses Cre recombinase under the control of the endogenous *Wnt1* promoter and enhancer, resulting in recombination in premigratory neural crest cells during embryonic development. Consequently, deletion of *Bdnf* occurs selectively in neural crest-derived tissues, including peripheral autonomic neurons and the intrinsic cardiac nervous system. Experimental knockout mice were homozygous for the floxed *Bdnf* allele and positive for the *Wnt1*-Cre2 transgene (*Bdnf*^flox/flox^; *Wnt1*-Cre2), whereas littermate *Bdnf*^flox/flox^ mice lacking the Cre transgene served as controls throughout the study.

### 4.3. Echocardiographic Assessment

Cardiac structure and function were assessed by transthoracic echocardiography using a high-frequency ultrasound imaging system (Vevo 2100, FUJIFILM VisualSonics Inc., Toronto, ON, Canada) equipped with a 40 MHz linear-array transducer (MS550). Echocardiographic examinations were performed in conscious adult mice to avoid the confounding effects of anesthetics on heart rate and autonomic regulation. Animals were gently restrained without sedation and allowed to acclimatize to the imaging procedure before data acquisition. Two-dimensional B-mode and M-mode images were obtained from the parasternal short-axis view at the level of the papillary muscles. Left ventricular (LV) end-diastolic internal diameter (LVID;d), LV end-systolic internal diameter (LVID;s), interventricular septal thickness at end-diastole and end-systole (IVS;d and IVS;s), and LV posterior wall thickness at end-diastole and end-systole (LVPW;d and LVPW;s) were measured from M-mode recordings according to the recommendations of the American Society of Echocardiography for small animal imaging. Left ventricular ejection fraction (EF) and fractional shortening (FS) were automatically calculated using the VisualSonics v10.0.0 analysis software. Heart rate (HR) was determined from the M-mode tracings. All echocardiographic examinations and offline analyses were performed by an experienced operator blinded to genotype. For each parameter, measurements were averaged from at least three consecutive cardiac cycles.

### 4.4. Immunohistochemical Analysis

Mice were euthanized with isoflurane and exsanguinated by cardiac perfusion with phosphate-buffered saline (PBS) containing heparin (1 U/mL). We used a peristaltic pump for in situ perfusion at 10 mL/min with 30 mL PBS/heparin followed by 40 mL cold PFA. The heart was removed and immersion-fixed in 4% paraformaldehyde (PFA) in PBS at 4 °C for 24 h, followed by a 24 h incubation in PBS containing 20% sucrose. Cryoprotected tissue was frozen with dry ice and embedded in OCT compound (Ted Pella, Inc., Redding, CA, USA). Sections of 20 μm thickness were cut at −20 to −25 °C using a Leica CM3050S cryostat (Leica Microsystems Inc., Bannockburn, IL, USA) and collected on charged slides. After sections dried, slides were boxed, wrapped in aluminum foil, and stored at −20 °C until further processing. Slide-mounted tissue sections were brought to room temperature, and each was stained for specific neuronal markers following the protocol of fluorescence from previous studies (Hoard et al., 2008) [16] with slight modification. In summary, slides were washed 4 × 5 min with PBS (pH 7.3), incubated for 10 min in PBS containing 0.4% Triton X-100 and 0.5% bovine serum albumin (BSA), and blocked for 2 h in PBS containing 1% BSA, 0.4% Triton X-100, and 10% normal donkey serum (Jackson ImmunoResearch Laboratories, West Grove, PA, USA). Each section was then single- or triple-labeled overnight using primary antibodies listed in Table 2. The following day, the sections were washed 4 × 5 min with PBS, followed by a 10 min incubation in PBS containing 0.4% Triton X-100 and 0.5% BSA. To reduce background staining, a second blocking step was added before application of secondary antibodies. Species-specific donkey secondary antibodies conjugated to Alexa Fluor 488, 555, 594, or 647 (Jackson ImmunoResearch Laboratories) were applied at a (1:200) dilution in PBS containing 0.4% Triton X-100 and 1% BSA, and sections were incubated for 2 h before slides were washed 4 × 5 min with PBS. After final washes with PBS, cover glasses were applied with Citifluor (Ted Pella, Inc.) and sealed with clear nail polish. For the BDNF antibody, vendor references studies showed elimination of staining after shBDNF knockdown and in tamoxifen-inducible knockout mice. For the TrkB antibody, the vendor shows that only proteins of appropriate molecular mass occur in Western blots of brain lysate. No staining was observed in control sections incubated without primary antibodies.

Labeled sections were viewed and photographed using an Olympus BX41 microscope equipped with a Q-Color 3 digital camera. Select slides were evaluated further with a Leica TCS SP8 Confocal Microscope (Leica Microsystems Inc.). Confocal images were collected at a resolution of 1024 × 1024 using 488, 552 and 638 nm laser lines. Stacks spanned the tissue thickness, and figures were created from maximum-intensity projection (MIP) images of individual channels and merged images.

### 4.5. Langendorff-Perfused Isolated Heart

Hearts were collected from 12–16 week old C57BL6/j mice (from the Jackson Laboratory) as previously described [42,43]. Mice were heparinized with 500U minutes before the surgery. Mice were sacrificed via cervical dislocation, and then hearts were quickly excised and transferred to ice-cold Krebs–Henseleit buffer (118 mM NaCl, 25 mM NaHCO_3_, 11mM D-glucose, 4.7 mM KCl, 1.17 mM MgSO_4_, 1.18 mM KH_2_PO_4_, and 2.00 mM CaCl_2_). Aorta was cannulated in less than 1.5 min from the heart removal from the mouse chest and coronaries flushed out of blood with warmed (37 °C) K-H solution and transferred to a Langendorff apparatus for retrograde perfusion with Krebs–Henseleit buffer warmed to 37.5 ± 0.5 °C and gassed with 95% O_2_ and 5% CO_2_ at a rate of 3.5/4.5 mL/min via a high-precision peristaltic pump. Coronary perfusion pressure was kept at 80 ± 5 mmHg through a pressure chamber placed 100 cm above the heart. A water-filled latex balloon was inserted into the left ventricle to measure cardiac functions, including heart rate, LV developed pressure, diastolic pressure, the rate of left ventricular pressure development, and pressure decay via an analog–digital interface (MP160 Biopac System Inc., Goleta, CA, USA). After a 30 min stabilization period under constant Langendorff perfusion, baseline hemodynamic parameters were recorded. Hearts were then perfused with the pharmacological treatments described in the Results section via infusion through a side port connected to the perfusion line. Recombinant BDNF was administered at a final concentration of 10 nM, either alone or in combination with atropine (10 nM) or neostigmine (100 nM). Hemodynamic parameters, including heart rate (HR), left ventricular developed pressure (LVDevP), rate-pressure product (RPP), and maximal and minimal dP/dt, were continuously recorded throughout the infusion period and expressed as percentage change from baseline, unless otherwise specified.

### 4.6. Experimental Design and Statistical Analysis

The experimental unit was the individual mouse for all in vivo experiments and the individual isolated heart for all Langendorff perfusion studies. Sample sizes were determined *a priori* based on previous studies from our laboratory using the same experimental models, which consistently detected biologically relevant differences in cardiac chronotropy and function with comparable group sizes.

Animals were allocated to experimental groups according to genotype for comparisons between control and neural crest-specific BDNF knockout (ncBDNF KO) mice; therefore, randomization was not applicable for these experiments. For ex vivo pharmacological studies, isolated hearts were randomly assigned to the different treatment protocols. Animals were age-matched and housed under identical environmental conditions. Echocardiographic acquisition and analysis were performed by an operator blinded to genotype. Identical microscope acquisition settings were used for all immunofluorescence analyses, and Langendorff perfusion experiments were conducted under standardized conditions using identical stabilization periods, perfusion parameters, and drug administration protocols.

Inclusion and exclusion criteria were established *a priori*. Only healthy adult mice with confirmed genotype and successful completion of the experimental procedures were included in the study. For Langendorff experiments, only hearts exhibiting stable sinus rhythm and stable baseline hemodynamic parameters following the equilibration period were included in the analysis. No animals, isolated hearts, or data points were excluded after completion of the experiments, and no statistical methods were used to identify or remove outliers. The exact sample size for each experiment is reported in the corresponding figure legends and tables. Echocardiographic analyses were performed in 6 control and 6 ncBDNF KO mice (n = 12 total). Langendorff experiments included 4 vehicle-treated and 6 BDNF-treated hearts (Figure 5), 8 isolated hearts analyzed under repeated-measures conditions (Figure 6), and 4 neostigmine + BDNF-treated hearts analyzed under repeated-measures conditions (Figure 7). The total number of animals used for functional experiments was 34.

Statistical analyses were performed using GraphPad Prism 11.0.2 (GraphPad Software, San Diego, CA, USA). Data distribution was assessed using the Shapiro–Wilk normality test. Comparisons between two independent groups were performed using an unpaired two-tailed Student’s *t*-test, with Welch’s correction when variances were unequal. When data were not normally distributed, the non-parametric Mann–Whitney *U* test was used.

For the experiments shown in Figure 5, different isolated hearts were allocated to either the vehicle or BDNF treatment group. To account for baseline variability between preparations, all functional parameters were expressed as the percentage change from baseline, and the responses observed after 10 min of perfusion were compared between the two independent experimental groups using the appropriate parametric or non-parametric test, according to data distribution.

Repeated measurements obtained from the same isolated hearts under different experimental conditions (Figure 6 and Figure 7) were analyzed using the non-parametric Friedman repeated-measures analysis of variance, followed by Dunn’s multiple-comparisons test when appropriate. Exact sample sizes, effect sizes, and 95% confidence intervals are reported in the corresponding figure legends and summarized in Supplementary Table S1. All statistical tests were two-tailed, and a *p* value < 0.05 was considered statistically significant.

## Figures and Tables

**Table 1:** Echocardiographic assessment of cardiac structure and function in control and neural crest-specific BDNF knockout mice.

Parameter	Control (Mean ± SD)	ncBDNF KO (Mean ± SD)	*p*-Value
HR	643.00 ± 28.90	686.44 ± 17.39	0.0132
IVS;d	0.92 ± 0.08	0.97 ± 0.15	0.5625
IVS;s	1.48 ± 0.12	1.46 ± 0.13	0.8010
LVID;d	2.85 ± 0.04	2.86 ± 0.17	0.8187
LVID;s	1.47 ± 0.22	1.44 ± 0.25	0.7718
LVPW;d	1.04 ± 0.11	1.02 ± 0.11	0.7184
LVPW;s	1.40 ± 0.07	1.46 ± 0.08	0.2741
EF	81.37 ± 7.74	82.53 ± 8.13	0.6696
FS	48.60 ± 7.75	49.81 ± 7.45	0.6706

Quantitative echocardiographic parameters obtained from adult control (n = 6) and neural crest-specific BDNF knockout (ncBDNF KO; n = 6) mice. Neural crest-specific BDNF deletion was associated with a significant increase in heart rate (HR), whereas interventricular septal thickness (IVS), left ventricular internal diameter (LVID), left ventricular posterior wall thickness (LVPW), ejection fraction (EF), and fractional shortening (FS) were not significantly different between groups, indicating preserved cardiac morphology and systolic performance. Statistical comparisons were performed using unpaired two-tailed Student’s *t*-test (Welch’s correction where appropriate). *p* < 0.05 was considered statistically significant.

**Table 2:** Primary antibodies.

Antibody	Host	Immunogen	Company	Catalog No.	RRID	Dilution
Anti-TrkB	goat	Mouse myeloma cell line NS0-derived recombinant mouse TrkB.	R&D Systems	AF1494	RRID: AB_2155264	1:200
Anti-BDNF	Rabbit	Peptide (C)VLEKVPVSKQLK, corresponding to amino acid residues 166–178 of human BDNF.	Alomone Labs	ANT-010	RRID: AB_2039756	1:200
Anti-Synaptophysin 1	chicken	Synthetic peptide corresponding to AA 301 to 313 from human Synaptophysin 1.	Synaptic Systems	101 006	RRID: AB_2622239	1:500
Anti-S100	rabbit	S100 isolated from cow brain.	Dako	GA50461-2	RRID: AB_2811056	1:400
Anti-Vesicular Acetylcholine Transporter (VAChT)	rabbit	Recombinant protein corresponding to AA 475 to 530 from rat VAChT.	Synaptic Systems	139 103	RRID: AB_887864	1:500
Anti-MAP2	chicken	Recombinant fragment corresponding to Human MAP2. Mix of recombinant human constructs of projection domain sequences, amino acids 235–1588.	abcam	ab5392	RRID: AB_2138153	1:1000

## Data Availability

The original contributions presented in this study are included in the article/Supplementary Materials. Further inquiries can be directed to the corresponding author.

## Supplementary Materials

The following supporting information can be downloaded at: https://www.mdpi.com/article/10.3390/ijms27167403/s1.
